# Specific Heat-Killed Lactic Acid Bacteria Enhance Mucosal Aminopeptidase N Activity in the Small Intestine of Aged Mice

**DOI:** 10.3390/ijms26125742

**Published:** 2025-06-15

**Authors:** Takeshi Tsuruta, Mami Wakisaka, Takumi Watanabe, Aoi Nishijima, Akihito Ikeda, Mao Teraoka, Tianyang Wang, Kuiyi Chen, Naoki Nishino

**Affiliations:** 1 Faculty of Environmental, Life, Natural Science and Technology, Okayama University, Okayama 7008530, Japan; prpt5ih3@s.okayama-u.ac.jp (M.W.); nishijima976@gmail.com (A.N.); nikumaki200@gmail.com (A.I.); mao.teraoka@s.okayama-u.ac.jp (M.T.); pvh11tk5@s.okayama-u.ac.jp (T.W.); pfiw96jr@s.okayama-u.ac.jp (K.C.); j1oufeed@okayama-u.ac.jp (N.N.); 2 Research Center for Intestinal Health Science, Okayama University, Okayama 7008530, Japan; 3 Bio-Lab Co., Ltd., Hidaka-shi 350-1249, Japan; t.watanabe@bio-ken.jp

**Keywords:** aging, aminopeptidase N, bone metabolism, lactic acid bacteria, small intestine

## Abstract

Aminopeptidase N (APN), an enzyme expressed in the small intestinal mucosa, is involved in dietary protein digestion. Previous studies have shown that oral administration of fermented milk containing lactic acid bacteria (LAB) enhances mucosal APN activity in young mice. This study aimed to investigate whether LAB strains stimulate mucosal APN activity in aged mice and to evaluate its relevance to age-related changes in body composition. The underlying molecular mechanisms were also explored in vitro. Experiment 1: Aged C57BL/6J mice were fed diets supplemented with heat-killed LAB strains—*Enterococcus faecalis* OU-23 (EF), *Leuconostoc mesenteroides* OU-03 (LM), or *Lactiplantibacillus plantarum* SNK12 (LP). Compared to the aged Control group, the ileal APN activity was significantly higher in the LP group. LP administration also elevated serum Gla-osteocalcin levels and decreased serum CTX-1 levels. Experiment 2: IEC-6 cells were co-cultured with LP that had been treated with RNase, DNase, or lysozyme. APN activity was significantly lower in cells co-cultured with DNase- or lysozyme-treated LP compared to those co-cultured with untreated LP. A specific LAB strain may enhance mucosal APN activity in the aged intestine, potentially contributing to improved bone metabolism. This effect may be mediated by bacterial DNA and peptidoglycan.

## 1. Introduction

Aging is characterized by a progressive loss of structure and function in various tissues and organs, leading to increased vulnerability to diseases [1,2,3]. In the small intestine, aging induces several morphological changes, such as thickened muscular layers, distorted villi, and an increased number of secretory Paneth and goblet cells [4,5,6,7,8,9,10], along with impaired digestive and absorptive functions. Using senescence-accelerated mouse strains, Suzuki et al. revealed that aged groups exhibited lower mRNA expression of genes related to the digestion and absorption of carbohydrates, peptides, amino acids, and lipids in the jejunum and ileum compared to young mice [11]. In inbred mouse strains such as Balb/c and C57BL/6 mice, the activity of brush-border membrane enzymes including aminopeptidase N (APN) declines with age [12,13]. APN plays a vital role in protein digestion and is widely distributed on the brush border of small intestinal epithelial cells [14]. Although previous findings on whether aging impairs dietary protein absorption have been inconsistent [15], an age-related decline in APN activity may potentially affect the absorption of dietary protein.

Interestingly, Thoreux et al. demonstrated that oral administration of fermented milk produced by *Lactobacillus casei* DN-114 001 increases APN activity in the brush border of the small intestine in young mice [16]. This report raises the possibility that stimulation of small intestinal epithelial cells by lactic acid bacteria (LAB) may enhance APN activity and help ameliorate the age-related decline in APN activity in the small intestine. However, the stimulatory effect of LAB on APN activity in the small intestine has not been evaluated in aged animals. It also remains to be determined whether restoring APN activity in the small intestine during aging can mitigate the decline in protein anabolic functions, including osteogenesis, albumin production, and skeletal muscle formation. Moreover, the molecular mechanisms by which LAB stimulate APN activity in small intestinal epithelial cells remain unclear. In the present study, we conducted the following two experiments to investigate these issues: (Experiment 1) Three species of heat-killed LAB were orally administered to aged mice for 3 months under sedentary conditions. This experiment aimed to assess whether LAB can stimulate membrane APN activity in the small intestine of aged animals, and to examine how such stimulation affects bone metabolism, skeletal muscle mass, and the biosynthesis of circulating proteins, including immunoglobulins and albumin. Heat-killed, rather than live, LAB were used to specifically evaluate the stimulatory effects of bacterial cell components. (Experiment 2) To further elucidate the mechanism underlying APN activation, the LAB species that exhibited the highest stimulatory effect in Experiment 1 was selected. The bacterial components responsible for enhancing APN activity were identified through in vitro assays using intestinal epithelial cell line IEC-6 cells.

## 2. Results

### 2.1. Experiment 1

#### 2.1.1. Body Weight, Skeletal Muscle and Adipose Tissue Weights, and Feed Intake

Final body weight, weights of the gastrocnemius muscle and epididymal adipose tissue, and average feed intake are summarized in Table 1. No significant differences in final body weight were observed among the groups. Similarly, no significant differences were detected in the weights of the gastrocnemius muscle or epididymal adipose tissue among the groups. Average feed intake tended to be higher in the LM (*p* = 0.07) and LP (*p* = 0.05) groups compared to the AC group, although these differences were not statistically significant.

#### 2.1.2. Total Length, Villous Height, and Mucosal APN Activity of Small Intestine

**Figure 1:**
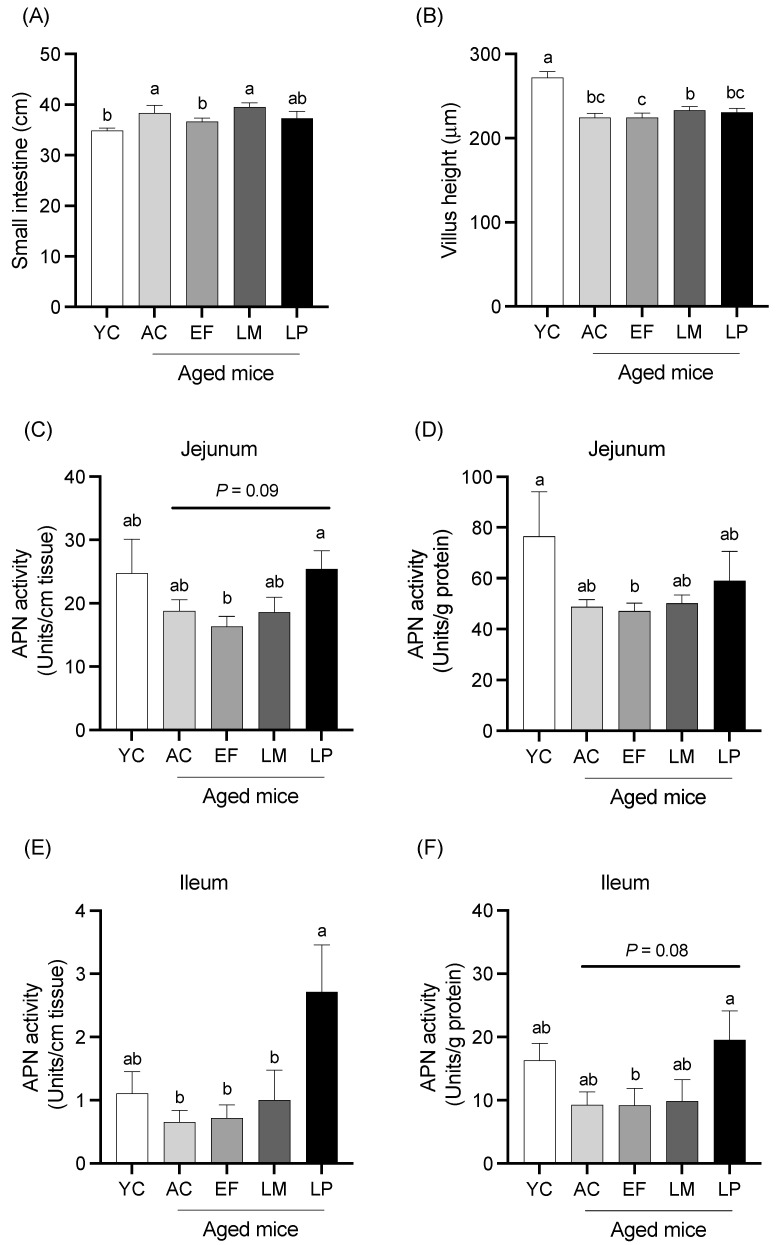
Length, villous height, and mucosal APN activity of the small intestine. (**A**) Small intestinal length; (**B**) villous height; and APN activity in the jejunum (**C**,**D**) and ileum (**E**,**F**), expressed per tissue length (units/cm) and protein content (units/g protein). Data are presented as mean ± SEM (*n* = 6 per group). Following the analysis of variance by Bartlett’s test, data were analyzed by a two-tailed one-way ANOVA (equal variances) or the Kruskal–Wallis test (unequal variances), and then by post hoc multiple comparisons tests, as needed. Values without a shared letter indicate statistically significant differences (*p* < 0.05). Data are representative of two independent experiments. Abbreviations: YC: young mice fed a Control diet; AC: aged mice fed a Control diet; EF: aged mice fed an *Enterococcus faecalis*-supplemented diet; LM: aged mice fed a *Leuconostoc mesenteroides*-supplemented diet; LP: aged mice fed a *Lactiplantibacillus plantarum*-supplemented diet; APN: aminopeptidase N.

The total length and average villous height of the small intestine are presented in Figure 1A,B. The total intestinal length in the EF group was significantly shorter than that in the AC group. The average villous height in all aged groups was significantly shorter than the YC group. Mucosal APN activity in the jejunum and ileum, expressed per unit length (U/cm tissue) and per unit protein (U/g protein), are shown in Figure 1C–F. In the jejunum, APN activity per unit length was significantly higher in the LP group than in the EF group and showed a trend toward being higher than in the AC group (*p* = 0.09) (Figure 1C). APN activity per unit protein was significantly lower in the EF group and showed a decreasing trend in the AC group relative to the YC group. No significant differences were observed in the LM and LP groups compared to the YC group (Figure 1D). In the ileum, APN activity per unit length was significantly higher in the LP group compared to the AC, EF, and LM groups (Figure 1E). APN activity per unit protein was significantly higher in the LP group than in the EF group and tended to be higher than in the AC (*p* = 0.08) and LM (*p* = 0.07) groups (Figure 1F).

#### 2.1.3. Serum L-Amino Acid and Total Protein Levels

**Figure 2:**
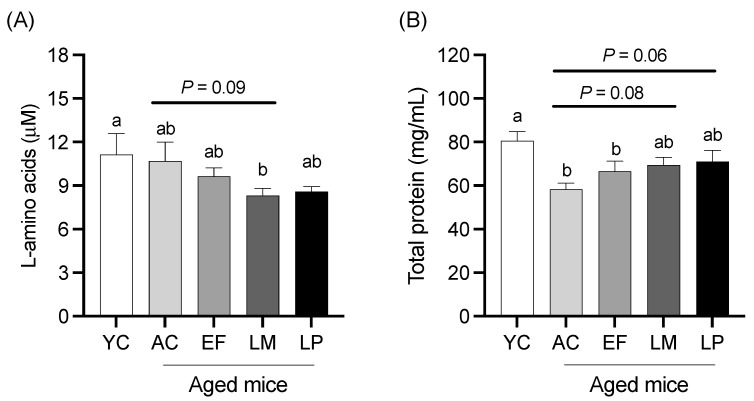
(**A**) L-amino acids and (**B**) total protein levels in serum. Data are presented as mean ± SEM (*n* = 6 per group). Following the analysis of variance by Bartlett’s test, data were analyzed by a two-tailed one-way ANOVA (equal variances) or the Kruskal–Wallis test (unequal variances), and then by post hoc multiple comparisons tests, as needed. Values without a shared letter indicate statistically significant differences (*p* < 0.05). Data are representative of two independent experiments. Abbreviations: YC: young mice fed a Control diet; AC: aged mice fed a Control diet; EF: aged mice fed an *Enterococcus faecalis*-supplemented diet; LM: aged mice fed a *Leuconostoc mesenteroides*-supplemented diet; LP: aged mice fed a *Lactiplantibacillus plantarum*-supplemented diet; APN: aminopeptidase N.

Serum L-amino acid and total protein levels are shown in Figure 2. The L-amino acid level in the LM group was significantly lower than that in the YC group and showed a tendency to be lower than that in the AC group (*p* = 0.09; Figure 2A). The total protein level in the LM and LP groups tended to be higher than that in the AC group (*p* = 0.08 and *p* = 0.06, respectively) (Figure 2B).

#### 2.1.4. BMD of Femur

**Figure 3:**
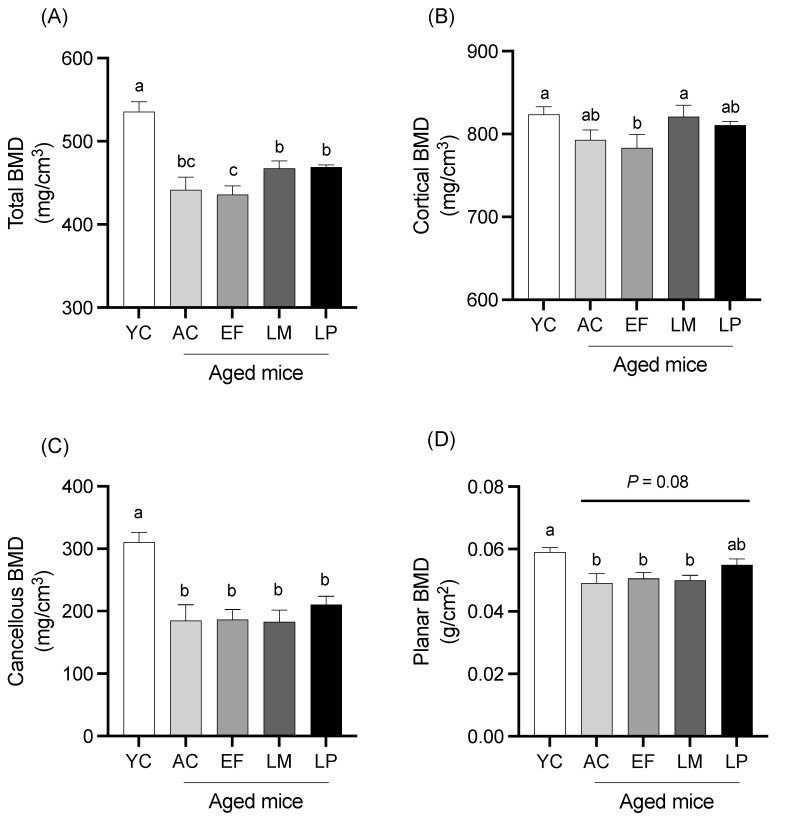
BMD of the femur. BMD of (**A**) total bone, (**B**) cortical bone, (**C**) cancellous bone, and (**D**) planar bone. Data are presented as mean ± SEM (*n* = 6 per group). Following the analysis of variance by Bartlett’s test, data were analyzed by a two-tailed one-way ANOVA (equal variances) or the Kruskal–Wallis test (unequal variances), and then by post hoc multiple comparisons tests, as needed. Values without a shared letter indicate statistically significant differences (*p* < 0.05). Data are representative of two independent experiments. Abbreviations: YC: young mice fed a Control diet; AC: aged mice fed a Control diet; EF: aged mice fed an *Enterococcus faecalis*-supplemented diet; LM: aged mice fed a *Leuconostoc mesenteroides*-supplemented diet; LP: aged mice fed a *Lactiplantibacillus plantarum*-supplemented diet; APN: aminopeptidase N.

BMD of total bone, cortical bone, cancellous bone, and planar bone in the femur is shown in Figure 3. Total BMD in the LM and LP groups was significantly higher than that in the EF group (Figure 3A). Cortical BMD in the LM group was also significantly greater than that in the EF group (Figure 3B). No significant differences in cancellous BMD were observed among the aged groups (Figure 3C). Planar BMD in the LP group showed a trend toward being higher than that in the AC group, although this did not reach statistical significance (*p* = 0.08) (Figure 3D).

#### 2.1.5. Serum Levels of Bone Metabolism Markers and Calcium

**Figure 4:**
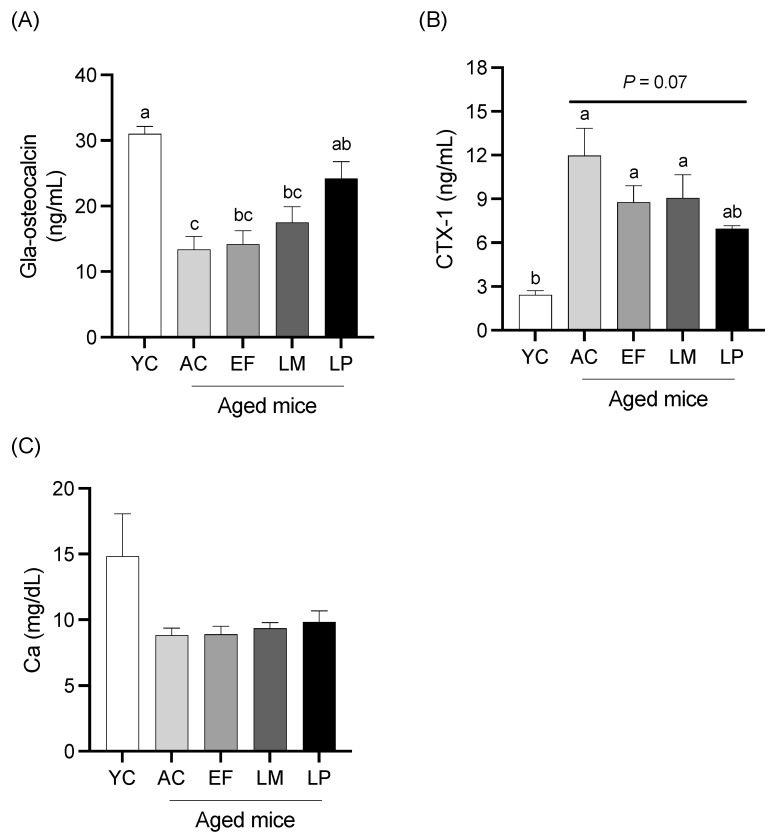
Bone-metabolism markers and calcium level in serum. (**A**) Gla-Osteocalcin, (**B**) CTX-1, and (**C**) calcium. Data are presented as mean ± SEM (*n* = 6 per group). Following the analysis of variance by Bartlett’s test, data were analyzed by a two-tailed one-way ANOVA (equal variances) or the Kruskal–Wallis test (unequal variances), and then by post hoc multiple comparisons tests, as needed. Values without a shared letter indicate statistically significant differences (*p* < 0.05). Data are representative of two independent experiments. Abbreviations: YC: young mice fed a Control diet; AC: aged mice fed a Control diet; EF: aged mice fed an *Enterococcus faecalis*-supplemented diet; LM: aged mice fed a *Leuconostoc mesenteroides*-supplemented diet; LP: aged mice fed a *Lactiplantibacillus plantarum*-supplemented diet; APN: aminopeptidase N.

Serum levels of Gla-Osteocalcin (a bone formation marker), CTX-1 (a bone resorption marker), and calcium are shown in Figure 4. Serum Gla-Osteocalcin levels in the AC, EF, and LM groups were significantly lower than that in the YC group, while the LP group exhibited significantly higher Gla-Osteocalcin levels compared to the AC group (Figure 4A). The serum CTX-1 levels in the AC, EF, and LM groups were significantly higher than that in the YC group. In contrast, the CTX-1 level in the LP group tended to be lower than in the AC group (*p* = 0.07) (Figure 4B). No significant differences in serum calcium levels were observed among the groups (Figure 4C).

### 2.2. Experiment 2

#### 2.2.1. APN Activity of IEC-6 Cells Co-Cultured with Heat-Killed LAB

**Figure 5:**
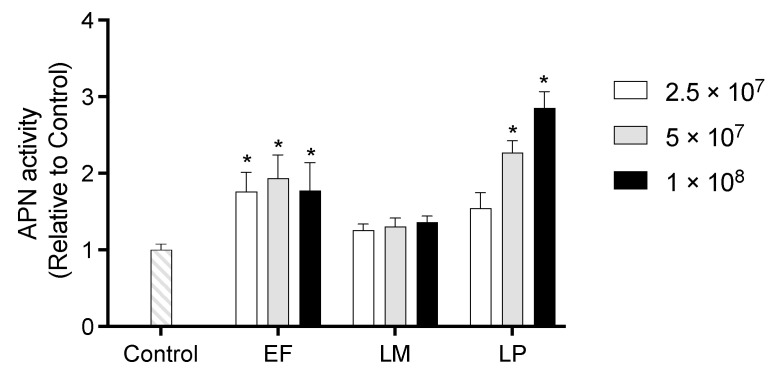
APN activity of IEC-6 cells co-cultured with heat-killed LAB at 2.5 × 10^7^, 5 × 10^7^, or 1 × 10^8^ cells per well. Data are representative of three independent experiments. Each experiment was conducted in quadruplicate. Data are presented as mean ± SEM. Following analysis of variance by Bartlett’s test, data were analyzed by two-tailed one-way ANOVA (equal variances) and then by post hoc multiple comparisons tests. * Statistically significant differences compared to Control (*p* < 0.05). Abbreviations: Control: cells without co-culture; EF: cells co-cultured with *Enterococcus faecalis*; LM: cells co-cultured with *Leuconostoc mesenteroides*; LP: cells co-cultured with *Lactiplantibacillus plantarum*; APN: aminopeptidase N.

APN activity of IEC-6 cells co-cultured with heat-killed LAB is shown in Figure 5. Cells treated with EF exhibited significantly higher APN activity compared to the Control cells. Additionally, co-culture with LP at 5 × 10^7^ or 1 × 10^8^ cells per well significantly increased APN activity relative to the Control cells, with the 1 × 10^8^ cells per well treatment yielding the highest activity among all treatment groups.

#### 2.2.2. APN Activity in IEC-6 Cells Co-Cultured with RNase-, DNase-, or Lysozyme-Treated LP

**Figure 6:**
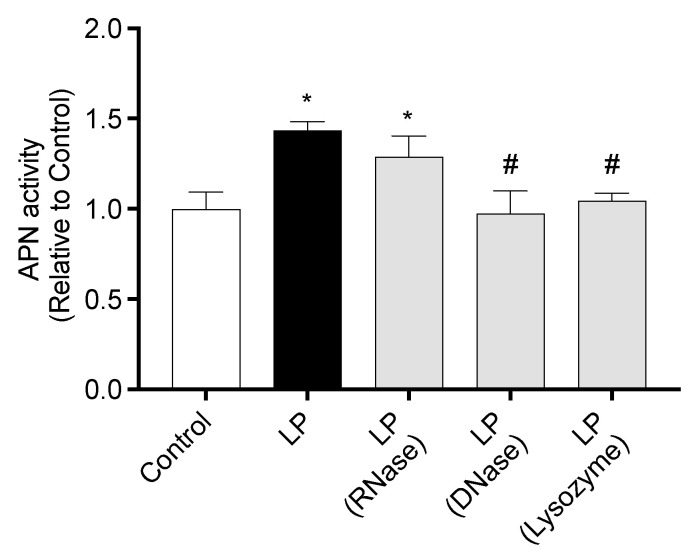
Effect of heat-killed *Lactiplantibacillus plantarum* cell component on APN activity of IEC-6 cells. APN activity of IEC-6 cells co-cultured with untreated, RNase-, DNase-, or lysozyme-treated *L. plantarum*. Data are representative of three independent experiments. Each experiment was conducted in quadruplicate. Data are presented as mean ± SEM. Following the analysis of variance by Bartlett’s test, data were analyzed by a two-tailed one-way ANOVA (equal variances) and then by post hoc multiple comparisons tests. Statistically significant differences compared to * Control or ^#^ LP (*p* < 0.05). Abbreviations: Control: cells without co-culture; LP: cells co-cultured with untreated *L. plantarum*; LP (RNase): cells co-cultured with RNase-treated *L. plantarum*; LP (DNase): cells co-cultured with DNase-treated *L. plantarum*; LP (Lysozyme): cells co-cultured with lysozyme-treated *L. plantarum*; APN: aminopeptidase N.

APN activity of IEC-6 cells co-cultured with RNase-, DNase-, or lysozyme-treated LP is shown in Figure 6. APN activity in cells co-cultured with untreated LP or RNase-treated LP was significantly higher than that in Control cells. In contrast, APN activity in cells co-cultured with DNase- or lysozyme-treated LP was significantly lower than that in cells co-cultured with untreated LP.

#### 2.2.3. APN Activity of IEC-6 Cells Treated with TLR2 Agonists

**Figure 7:**
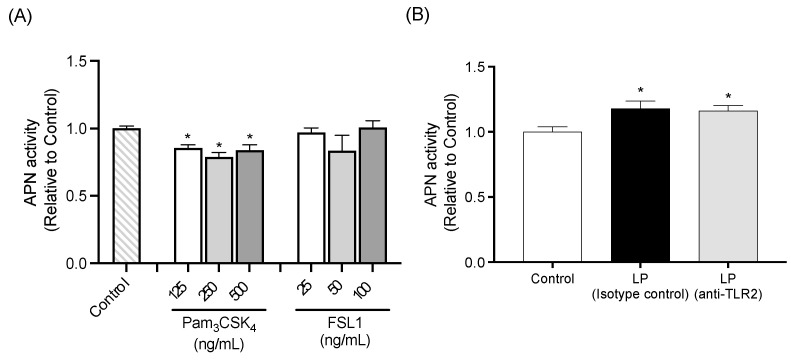
Influence of TLR2 signaling on APN activity of IEC-6 cells. (**A**) APN activity in cells treated with TLR2 agonists including Pam_3_CSK_4_ at 125, 250, or 500 ng/mL and FSL1 at 25, 50, or 100 ng/mL. (**B**) Effect of TLR2 neutralization on APN activity in cells co-cultured with *L. plantarum*. Data are representative of two independent experiments. Each experiment was conducted in quadruplicate. Data are presented as mean ± SEM. Following analysis of variance by Bartlett’s test, data were analyzed by a two-tailed one-way ANOVA (equal variances) and then by post hoc multiple comparisons tests. Statistically significant differences compared to * Control (*p* < 0.05). Abbreviations: Control: cells without co-culture and treatment; LP (Isotype control): cells treated with isotype control antibody and co-cultured with *L. plantarum*; LP (anti-TLR2): cells treated with anti-TLR2 antibody and co-cultured with *L. plantarum*; APN: aminopeptidase N.

APN activity of IEC-6 cells treated with TLR2 agonists is shown in Figure 7A. Cells treated with Pam_3_CSK_4_ exhibited significantly lower APN activity compared to the Control cells, regardless of Pam_3_CSK_4_ concentration. No significant differences in APN activity were observed between Control cells and cells treated with FSL1.

#### 2.2.4. TLR2 Neutralization Study

APN activity was significantly higher in cells treated with heat-killed LP in conjunction with either isotype control antibody or TLR2 neutralizing antibody, compared to the Control cells. There was no significant difference in APN activity between the two antibody treatment groups (Figure 7B).

## 3. Discussion

In the present study, we sought to confirm whether the intake of specific LAB stimulates membrane APN activity in the small intestine of aged animals, which contributes to the amelioration of aging-induced bone loss and reduction in skeletal muscle mass and circulating protein levels. We observed that oral administration of LP to aged mice increased APN activity in the small intestine, particularly in the ileum, compared to the AC group, despite no significant differences in small intestinal length or villous height between the LP group and other aged groups. In contrast, no increase in APN activity was observed in the EF and LM groups. These findings suggest that LP may enhance APN activity independently of intestinal surface area or length, and that the effect of LAB on small intestinal APN activity may vary depending on the bacterial species. Previous studies have demonstrated that a phylogenetic analysis of *Lactobacillales* based on concatenated DNA-dependent RNA polymerase subunit sequences reveals distinct evolutionary relationships among *E. faecalis*, *L. mesenteroides*, and *L. plantarum* [17]. Furthermore, the structure of PGN also differs among these species [18,19,20]. Differences in bacterial components, such as genomic DNA sequences and PGN structure, may underlie the species-specific stimulatory effects of LAB on APN activity.

Serum L-amino acid levels tended to be lower in the LM and LP groups compared to the other groups. Furthermore, levels of non-esterified fatty acids (NEFA) and ketone bodies, metabolic indicators of energy status, and urea nitrogen, an indicator of amino acid oxidation, did not significantly differ between the LM or LP groups and the AC group (Table S1). These findings suggest that the reduced serum L-amino acid levels observed in the LM and LP groups are unlikely to result from negative energy balance or increased amino acid catabolism. Notably, LP intake tended to attenuate the age-related decline in total serum protein and significantly attenuated the decline in Gla-osteocalcin levels, implying that LP may stimulate protein anabolic processes. Castellino et al. previously demonstrated that insulin-driven protein anabolism reduces circulating amino acid concentrations, particularly via enhanced leucine uptake and incorporation into muscle protein [21]. These findings collectively suggest that LP-induced protein anabolism may be associated with a transient reduction in circulating L-amino acid levels.

The LP group also exhibited a trend toward increased planar BMD in the femur relative to the AC group. In addition, LP administration elevated serum Gla-osteocalcin levels, a marker of bone formation, and tended to decrease serum CTX-1 levels, a marker of bone resorption, suggesting that LP may counteract age-associated bone loss by modulating bone metabolism. Islamoglu et al. reported that lower dietary protein intake is associated with reduced osteocalcin and elevated N-terminal telopeptide of type I collagen (NTX), a bone resorption marker, in postmenopausal women [22]. Taken together, our findings indicate that LP may improve bone metabolism by promoting dietary protein absorption. However, whether the elevated APN activity observed in the LP group contributes to this improvement remains unclear, and further investigation is required.

Despite the notable effect of LP on bone metabolism, no significant differences in skeletal muscle mass were observed among the aged groups. Given that muscle hypertrophy typically requires mechanical loading [23,24], the absence of muscle growth in the current study may be attributed to insufficient physical stimulus under sedentary conditions. Future studies should assess the effect of LP intake on skeletal muscle development in conjunction with mechanical loading.

In vitro experiments using IEC-6 cells were conducted to elucidate the underlying mechanisms by which LP stimulates APN activity in small intestinal epithelial cells. Consistent with the findings from the animal study, LP treatment significantly enhanced APN activity in IEC-6 cells in a dose-dependent manner. In contrast to the in vivo results, however, EF treatment also increased APN activity in IEC-6 cells, suggesting that the stimulatory effects of LAB on APN activity may differ between mice and rats.

Notably, treatment of LP with DNase abolished the LP-induced APN activity in IEC-6 cells. Previous studies have reported that bacterial DNA derived from probiotics exerts various physiological effects on intestinal epithelial cells. For instance, Jijon et al. demonstrated that DNA extracted from the probiotic mixture VSL#3 suppressed TNF-α-induced proinflammatory responses in HT-29 cells. Furthermore, Song et al. reported that oral administration of DNA isolated from *Lactobacillus rhamnosus* GG upregulated programmed death-ligand 1 expression in jejunal epithelial cells, thereby promoting apoptosis of type 2 helper T cells. Given the limited capacity for DNA uptake in intact small intestinal epithelial cells, it is assumed that LP DNA may be recognized by cell surface receptors rather than cytosolic sensors such as TLR9 or STING. However, the mechanisms by which intestinal epithelial cells recognize bacterial DNA via the cell surface receptors remain poorly understood. Likewise, treatment of LP with lysozyme, a muramidase that hydrolyzes the β-1,4-glycosidic bond between N-acetylmuramic acid (MurNAc) and N-acetylglucosamine (GlcNAc) in peptidoglycan (PGN) [25], abolished APN activity induced by LP, suggesting that the intact PGN structure is essential for its stimulatory effect. We further evaluated the involvement of TLR2, a known receptor for PGN, in this process. However, neither Pam_3_CSK_4_ nor FSL1 (TLR2 agonists) activated APN in IEC-6 cells. Moreover, a TLR2-neutralizing antibody failed to attenuate LP-induced APN activation. These observations indicate that LP-mediated stimulation of APN activity may involve TLR2-independent signaling pathways. Despite maintaining Gram-positive PGN integrity (Figure S1C), DNase-treated LP failed to induce APN activity in IEC-6 cells. Additionally, DNA extracted from LP alone did not stimulate APN activity (Figure S2), nor did lysozyme-treated LP, which retained similar DNA content (Figure S1B). Taken together, these findings suggest that both the intact PGN structure and bacterial DNA may be required for LP-induced APN activation in small intestinal epithelial cells, as neither PGN nor DNA alone was sufficient to activate APN. To further substantiate this hypothesis, additional studies are required to evaluate whether APN is activated by PGN alone or in combination with DNA extracted from LP.

We acknowledge several limitations in the present study. First, the relatively small sample size (*n* = 6) used in this study represents a limitation, particularly in the analysis of APN activity in the small intestine and bone-related parameters, where some results showed only statistical trends without reaching significance. Further studies with larger sample sizes will be essential to validate and extend these results. Second, this study evaluated the effects on APN activity using only three strains of heat-killed LAB. Future research should involve large-scale screening using additional LAB strains, as well as investigations into the potential synergistic effects of combinations of multiple strains. Third, heat-killed LAB were used to specifically evaluate the stimulatory effects of bacterial cell components. Therefore, the impact of LAB-derived metabolites, such as organic acids, on APN activity in the aged intestine remains unclear. Previous studies have shown that the physiological activities of LAB differ between heat-killed and live forms [26]. Comparative studies using both heat-killed and live LAB are warranted to clarify the respective contributions of bacterial cell components and metabolites.

Accumulating evidence suggests that an adequate supply of amino acids derived from dietary protein is one of the key factors in preventing osteoporosis. Osteoporosis is a systemic skeletal disease, particularly prevalent among the elderly, characterized by reduced bone mass and deterioration of bone microarchitecture, ultimately leading to increased bone fragility and fracture risk [27,28]. A cross-sectional study of middle-aged and older adults in the U.S. conducted by Zhang et al. reported a significant association between low protein intake and an elevated risk of osteoporosis and hip fracture [29]. Similarly, mice fed a low-protein diet exhibited significantly lower BMD in the femur and lumbar spine compared to those provided with adequate protein intake [30]. Although several drugs for the treatment of osteoporosis have been developed—such as vitamin D analogs, calcium supplements, and antiresorptive agents—their adverse side effects remain a major concern. Therefore, there is an urgent need to identify and develop novel therapeutic strategies for osteoporosis that are both effective and have minimal side effects. Enhancement of APN activity in the small intestinal mucosa through stimulation by bacterial cells of LAB may serve as a potential strategy for preventing osteoporosis.

## 4. Materials and Methods

### 4.1. Experiment 1

#### 4.1.1. Preparation of Heat-Killed Lactic Acid Bacteria and Diet

Three strains of LAB, namely *Enterococcus faecalis* OU-23 (EF), *Leuconostoc mesenteroides* OU-03 (LM), and *Lactiplantibacillus plantarum* SNK12 (LP), were obtained from Bio-Lab Co., Ltd. (Saitama, Japan). Each LAB strain was aerobically cultured overnight at 37 °C in MRS broth (Difco, Detroit, MI, USA), washed with distilled water, and centrifuged at 10,000× *g* for 3 min. The bacterial suspension in distilled water [20–30 mg (wet bacteria weight)/mL] was heat-treated at 105 °C for 30 min using an autoclave (HV-25IILB; Hirayama Manufacturing Corp., Saitama, Japan). The heating temperature was selected to avoid the destruction of the bacterial cell wall. The inactivation of LAB was confirmed using plate count agar with bromocresol purple (Nissui Pharmaceutical, Tokyo, Japan). The heat-killed LAB were subsequently processed with a high-pressure homogenizer (ECONIZER LABO-01; Sanmaru Machinery Co., Ltd., Shizuoka, Japan) at 15 MPa. An equal amount of dextrin (NSD300; Sanei Sucrochemical Co., Ltd., Aichi, Japan) was added to the homogenized bacteria, and the mixture was dried into powder using a spray dryer (ADL311S-A; Yamato Scientific Co., Ltd., Tokyo, Japan). Both Control and LAB-supplemented pellet diets were procured from CLEA Japan (Tokyo, Japan). The Control diet composition was based on the AIN93G. Each heat-killed LAB was added to the diet at 2 g (1 × 10^12^ cells)/kg by replacing maize starch. The amount of LAB used in the diets was determined based on a previous study [31,32]. The actual compositions of the experimental diets are shown in Table 2.

#### 4.1.2. Animal Experiment

Male C57BL/6J mice, either young (5 weeks old) or aged (70 weeks old), were obtained from Jackson Laboratory Japan (Kanagawa, Japan). Throughout the study, animals were maintained in a temperature-controlled environment with a 12 h light/dark cycle and given free access to food and water. Following a 7-day acclimation period, aged mice were weighed and randomly assigned to four groups (*n* = 6 per group) to ensure similar average body weights across groups. Experimental groups were as follows: young mice fed a Control diet (YC group); aged mice fed a Control diet (AC group); aged mice fed an EF-supplemented diet (EF group); aged mice fed an LM-supplemented diet (LM group); and aged mice fed an LP-supplemented diet (LP group). During the 12-week intervention, mice were housed two per cage. Feed intake per cage was recorded weekly, and the average daily intake per mouse was calculated accordingly. At the end of the feeding period, mice were euthanized by CO_2_ narcosis. Cardiac blood was collected via heart puncture, and the serum was separated by centrifugation at 1,500× *g* for 15 min at 4 °C. During necropsy, the small intestine was removed and measured from the pylorus to the ileocecal junction. After excising the duodenum (first 3 cm), the remaining small intestine was evenly divided into two segments designated as jejunum and ileum. The jejunoileal junction was fixed in Yufix (Sakura Finetek Japan, Tokyo, Japan) for hematoxylin and eosin (HE) staining. Ten centimeters of both jejunal and ileal segments were collected for APN activity assays. Additionally, the epididymal adipose tissue and gastrocnemius muscles were harvested and weighed. The femur bones were collected for bone mineral density (BMD) analysis.

#### 4.1.3. APN Activity in Small Intestinal Mucosa

The jejunal and ileal segments were longitudinally incised, and the mucosal layer was isolated by gentle scraping using a glass slide. The collected mucosal scrapings were suspended in 500 μL of 50 mM phosphate buffer (pH 7.0), homogenized with a homogenizer (Model S-203; Ikedarika Co. Ltd., Tokyo, Japan) at minimum speed for 15 s, and centrifuged at 12,000× *g* for 10 min at 4 °C. The resulting supernatant was collected and used for the measurement of APN (EC 3.4.11.2) activity. APN activity was assessed using a previously described method with minor modifications [33]. Briefly, 50 μL of the mucosal supernatant was incubated at 37 °C for 30 min and subsequently mixed with a pre-warmed substrate solution composed of 50 mM phosphate buffer (pH 7.0) and 10 mM L-alanine-*p*-nitroanilide hydrochloride (Sigma-Aldrich Japan, Tokyo, Japan). After incubation at 37 °C for 1 h, the absorbance at 405 nm was measured using a microplate reader (Model SH-9000; Hitachi High-Technologies, Tokyo, Japan) to quantify the amount of *p*-nitroaniline (pNA) released by the enzymatic activity of APN. Serial dilutions of a pNA standard solution (Tokyo Chemical Industry, Tokyo, Japan) were also measured to generate a standard curve, which was subsequently used to calculate the pNA concentrations in the samples. One unit of APN activity was defined as the amount of enzyme that catalyzes the release of 1 nmol of pNA per minute. The protein concentration in the mucosal supernatant was determined using a BCA Protein Assay Kit (TaKaRa Bio Inc., Shiga, Japan) according to the manufacturer’s instructions. Enzyme activity was expressed as APN units per centimeter of intestinal tissue (nmol/min/cm tissue) and per gram of mucosal protein (nmol/min/g protein).

#### 4.1.4. Measurement of Small Intestinal Villus Height

The fixed jejunoileal tissue was dehydrated, embedded in paraffin, and sectioned at a thickness of 5 μm. For histological evaluation, tissue sections were stained using the standard hematoxylin and eosin protocol. Villus height was assessed in cross sections examined under 200× magnification using an all-in-one fluorescence microscope (BZ-X710; Keyence, Osaka, Japan). Five images per mouse were captured, and villus height was blindly measured in eight well-oriented villi per image using ImageJ software (version 1.8.0).

#### 4.1.5. Micro-X-Ray-Computed Tomography Analysis of Femur

The BMD of the total bone, cortical bone, cancellous bone, and planar bone were measured by micro-X-ray-computed tomography (μCT) (Latheta LCT-200; Hitachi Aloka Medical, Tokyo, Japan). The average value was determined based on 96-μm-thick slices from a cross-section of the femur. Image data were quantified using an automated image analysis system (Latheta software ver. 2.10; Hitachi Aloka Medical).

#### 4.1.6. Serum Biochemical Analysis

The serum samples collected at necropsy were used for biochemical analyses. L-amino acid concentrations were determined using an L-Amino Acid Assay Kit (Colorimetric) (Cell Biolabs, San Diego, CA, USA). Total protein levels were measured with a BCA Protein Assay Kit (TaKaRa Bio Inc., Shiga, Japan). The concentrations of Osteocalcin containing two or three γ-carboxyglutamate residues (Gla-osteocalcin) and C-terminal telopeptides of type I collagen (CTX-1) were measured using enzyme-linked immunosorbent assays (ELISAs) with a Mouse Gla-Osteocalcin High Sensitive EIA Kit (TaKaRa Bio Inc.) and a Mouse CTX-1 ELISA Kit (Novus Biologicals, Littleton, CO, USA), respectively. Total calcium concentrations were determined using the Calcium E-Test Wako (FUJIFILM Wako, Osaka, Japan).

### 4.2. Experiment 2

#### 4.2.1. Cell Line

IEC-6 (RCB0993, Riken Cell Bank, Ibaraki, Japan) that originated from nontransformed rat intestinal crypt cells [34] was used as a model of intestinal membranous digestion of APN. Cells were maintained in Dulbecco’s modified Eagle’s medium (DMEM; FUJIFILM Wako) supplemented with 5% fetal bovine serum (FBS; GIBCO, Grand Island, NY, USA), penicillin (100 units/mL; FUJIFILM Wako), streptomycin (100 μg/mL; FUJIFILM Wako), and 4 µg/mL insulin (FUJIFILM Wako) in a humidified incubator in an atmosphere of 5% CO_2_ at 37 °C.

#### 4.2.2. APN Activity in IEC-6 Cells Co-Cultured with Heat-Killed LAB

The heat-killed LAB were washed with PBS to remove dextrin and resuspended in test media: DMEM supplemented with 1% FBS, penicillin (100 units/mL), streptomycin (100 μg/mL), and 4 µg/mL insulin. IEC-6 cells were seeded into a 96-well culture plate at a density of 4 × 10^3^ cells per well in the test medium. After 24 h of incubation, heat-killed LAB strains including EF, LM, and LP were added to the wells at final concentrations of 2.5 × 10^7^, 5 × 10^7^, or 1 × 10^8^ cells per well. The amount of LAB used in the cell culture experiments was determined based on a previous study [32]. An equal volume of test medium was added to the Control cells. Following a 48 h incubation period, APN activity was assessed as follows: the culture medium was removed, and each well was washed with 200 µL of PBS. After removing the PBS, 100 µL of substrate solution [50 mM phosphate buffer (pH 7.0) containing 10 mM L-alanine-*p*-nitroanilide hydrochloride] pre-warmed to 37 °C was added to each well. The plate was then incubated at 37 °C for 1 h, after which the absorbance at 405 nm was measured using a microplate reader. The amount of pNA released by APN was quantified using a standard curve prepared from serial dilutions of pNA. One unit of APN activity was defined as described in Section 2.1.4. To normalize APN activity based on viable cell number, the substrate solution was replaced with PBS, and 10 µL of Cell Counting Kit-8 reagent (CCK-8; Dojindo Laboratories, Kumamoto, Japan) was added to each well. After 1 h incubation, absorbance at 450 nm was measured using a microplate reader. APN activity in each well was normalized by dividing APN units by the corresponding CCK-8 absorbance value. APN activity in each treatment group was expressed as a relative value compared to that of the Control cells.

#### 4.2.3. APN Activity in IEC-6 Cells Co-Cultured with RNase-, DNase-, or Lysozyme-Treated LP

After washing with PBS, LP was treated with either 100 µg/mL RNase A (Nippon Gene, Toyama, Japan) in 10 mM Tris-HCl (pH 8.0) for 24 h, 100 units/mL DNase (Nippon Gene) in 1× DNase buffer (Nippon Gene) for 48 h, or 1.25 mg/mL lysozyme (FUJIFILM Wako) in 50 mM Tris-HCl, 10 mM MgCl_2_, and 0.25 M sucrose (pH 7.2) for 1 h at 37 °C. Following treatment, the bacterial cells were washed twice with 1 mL PBS and resuspended in the test medium. IEC-6 cells were seeded into 96-well culture plates at a density of 4 × 10^3^ cells per well in the test medium. After 24 h, untreated LP, RNase-treated LP, DNase-treated LP, or lysozyme-treated LP was added at 1 × 10^8^ cells per well. An equal volume of test medium was added to the Control cells. After 48 h of incubation, APN activity was measured as described in Section 4.2.5.

#### 4.2.4. APN Activity in IEC-6 Cells Treated with Toll-Like Receptor 2 Agonists

Pam_3_CSK_4_ and FSL1 (InvivoGen, San Diego, CA, USA), which are specific agonists of Toll-like receptor (TLR)2/TLR1 and TLR2/TLR6, were used to stimulate IEC-6 cells via their corresponding TLR pathways. IEC-6 cells were seeded into a 96-well culture plate at a density of 4 × 10^3^ cells per well in the test medium. After incubation for 24 h, cells were treated with FSL-1 at final concentrations of 25, 50, or 100 ng/mL, or with Pam_3_CSK_4_ at final concentrations of 125, 250, or 500 ng/mL. An equal volume of test medium was added to the Control cells. After incubation for 48 h, APN activity was determined as described in Section 4.2.5.

#### 4.2.5. TLR2 Neutralization Study

IEC-6 cells were seeded into 96-well culture plates at a density of 4 × 10^3^ cells per well in the test medium. After incubation for 24 h, cells were treated with a TLR2-neutralizing antibody (C9A12, InvivoGen) or an isotype control antibody (T9C6, InvivoGen) at a concentration of 10 µg/mL. After incubation at 37 °C for 30 min, heat-killed LP was added to each well at 1 × 10^8^ cells per well. An equal volume of test medium was added to the Control cells. After incubation for 48 h, APN activity was determined as described in Section 4.2.5.

#### 4.2.6. Statistical Analysis

Results are expressed as mean ± standard error of the mean (SEM). Statistical analyses were performed using Prism for Mac, version 10 (GraphPad Software, San Diego, CA, USA). The normality of data distribution was assessed using the Shapiro–Wilk test. For non-normally distributed data, group differences were analyzed using the Kruskal–Wallis test. For data that met the assumption of normality, homogeneity of variance was assessed using Bartlett’s test. Depending on the results of Bartlett’s test, either a one-way analysis of variance (ANOVA) or the Kruskal–Wallis test was employed. When overall significance was observed, post hoc multiple comparisons were conducted with false discovery rate correction using the Benjamini–Hochberg method. All statistical tests were two-tailed. A *p*-value < 0.05 was considered statistically significant, while a *p*-value between 0.05 and 0.1 was considered to indicate a statistical trend. Values without a shared letter indicate statistically significant differences (*p* < 0.05).

## 5. Conclusions

Our study demonstrates that oral administration of heat-killed LP enhances mucosal APN activity in the small intestine of aged mice, which is associated with improved bone metabolism and elevated circulating protein levels. In vitro assays using IEC-6 cells suggest that APN activation may be mediated by the synergistic stimulatory effects of DNA and PGN, both derived from heat-killed LP. These results imply that bacterial cells from a specific LAB strain can augment mucosal APN activity in the aged intestine. Furthermore, APN activity in small intestinal epithelial cells may serve as a useful selection criterion for identifying LAB strains that mitigate age-related disruption in bone remodeling and decline in systemic protein levels.

## Figures and Tables

**Table 1:** Body weight, skeletal muscle and adipose tissue weights, and feed intake.

	YC	Aged Mice			
		AC	EF	LM	LP
Final body weight (g)	41.17 ± 1.42	46.68 ± 3.23	45.63 ± 2.40	46.87 ± 2.85	45.48 ± 1.94
Gastrocnemius muscle					
Weight (g)	0.18 ± 0.00	0.15 ± 0.03	0.18 ± 0.00	0.17 ± 0.01	0.16 ± 0.01
Ratio in BW (%)	0.44 ± 0.01 ^a^	0.31 ± 0.06 ^b^	0.40 ± 0.02 ^ab^	0.36 ± 0.01 ^b^	0.35 ± 0.02 ^b^
Epididymal adipose tissue					
Weight (g)	1.07 ± 0.07	1.25 ± 0.20	1.38 ± 0.15	1.23 ± 0.15	1.30 ± 0.07
Ratio in BW (%)	2.61 ± 0.14	2.60 ± 0.31	3.01 ± 0.28	2.58 ± 0.24	2.88 ± 0.20
Feed intake (g/mouse/day)	2.26 ± 0.19	2.14 ± 0.23	2.27 ± 0.17	2.56 ± 0.24	2.51 ± 0.21

Values are given as means ± SEMs. Values without a common letter are statistically significantly different (*p* < 0.05). One-way ANOVA or Kruskal-Wallis test followed by post-hoc test was conducted. Abbreviations: YC: young mice fed a Control diet; AC: aged mice fed a Control diet; EF: aged mice fed an *Enterococcus faecalis*-supplemented diet; LM: aged mice fed a *Leuconostoc mesenteroides*-supplemented diet; LP: aged mice fed a *Lactiplantibacillus plantarum*-supplemented diet; APN: aminopeptidase N.

**Table 2:** Composition of experimental diets.

Ingredients (g/kg Diet)	Control Diet	LAB-Supplemented Diet
Maize starch	519.486	517.486
α- Maize starch	10	10
Sucrose	100	100
Casein	200	200
Soybean oil	70	70
Cellulose	50	50
AIN-93 Vitamin mix	10	10
AIN-93 Mineral mix	35	35
L-Cystine	3	3
Choline bitartrate	2.5	2.5
Tert-butylhydroquinone	0.014	0.014
Heat-killed LAB	-	2
Total	1000	1000

Abbreviations: LAB: lactic acid bacteria.

## Data Availability

The data that support the findings of this study, including the raw data underlying the graphs and tables, are available from the corresponding author upon reasonable request.

## Supplementary Materials

The supporting information can be downloaded at: https://www.mdpi.com/article/10.3390/ijms26125742/s1. Reference [35] is cited in the supplementary materials.

## Abbreviations

APN	Aminopeptidase N
ANOVA	Analysis of variance
BCA	Bicinchoninic acid
BMD	Bone mineral density
CCK-8	Cell counting kit-8
CTX-1	C-terminal telopeptides of type I collagen
EF	*Enterococcus faecalis*
FBS	Fetal bovine serum
FSL1	Fibroblast-stimulating lipopeptide1
GlcNAc	N-acetylglucosamine
HE	Hematoxylin and eosin
IEC-6	Intestinal epithelial cell 6
LAB	Lactic acid bacteria
LM	*Leuconostoc mesenteroides*
LP	*Lactiplantibacillus plantarum*
MurNAc	N-acetylmuramic acid
NEFA	Non-esterified fatty acids
NTX	N-terminal telopeptide of type I collagen
Pam_3_CSK_4_	Pam_3_Cys-Ser-Lys_4_
PGN	Peptidoglycan
pNA	*p*-nitroaniline
STING	Stimulator of interferon genes
TLR	Toll-like receptor
TNF-α	Tumor necrosis factor-α
