# Supplementary material for: Specific Heat-Killed Lactic Acid Bacteria Enhance Mucosal Aminopeptidase N Activity in the Small Intestine of Aged Mice
Source: Int J Mol Sci. 2025 Jun 15;26(12):5742. doi: 10.3390/ijms26125742 (PMC12193318; doi:10.3390/ijms26125742)
Supplement: Supplementary file 1 [file ijms-26-05742-s001.zip › ijms-3682111-supplementary.pdf]

Supplementary Materials

International Journal of Molecular Sciences

Title: Specific heat-killed lactic acid bacteria enhance mucosal aminopeptidase N activity in the small intestine of aged mice

Takeshi Tsuruta [1,2,§,*], Mami Wakisaka [1,§], Takumi Watanabe [3], Aoi Nishijima [1], Akihito Ikeda [1], Mao Teraoka [1], Tianyang Wang [1] Kuiyi Chen [1] and Naoki Nishino [1,2]

[1] Faculty of Environmental, Life, Natural Science and Technology, Okayama University, Okayama 700-8530, Japan

[2] Research Center for Intestinal Health Science, Okayama University, Okayama 700-8530, Japan

[3] Bio-Lab Co., Ltd., Hidaka, Saitama, Japan

[§]These authors contributed equally to the work.

*Corresponding author: Faculty of Environmental, Life, Natural Science and Technology, Okayama University, 1-1-1 Tsushima-Naka, Kita-ku, Okayama 700-8530, Japan

Tel/Fax: +81-86-251-8336

E-mail address: tsurutafe@okayama-u.ac.jp

1. Materials and Methods

1.1. Serum biochemical analysis

Serum samples collected at necropsy were used for biochemical analyses. Levels of immunoglobulin G (IgG), albumin, non-esterified fatty acid (NEFA), ketone bodies, and urea nitrogen were quantified using the following commercial kits, in accordance with the manufacturers' instructions: mouse IgG ELISA kit (Bethyl Laboratories, Montgomery, TX, USA), L-type Wako ALB-BCP, LabAssay NEFA, Autowako Total Ketone Body Assay Kit, and L-type Wako UN kit (FUJIFILM Wako, Osaka, Japan).

1.2. Nucleic acids concentration and Gram staining of enzyme-treated *Lactobacillus plantarum*

Total RNA and DNA were extracted from untreated *L. plantarum* (LP), as well as from RNase-, DNase-, or lysozyme-treated LP, using ISOGEN (Nippon Gene). Briefly, the bacterial cells were resuspended in 500 μL of ISOGEN containing 200 mg of zirconia beads and homogenized using a bead beater-type homogenizer (Beads Crusher μT-12; TAITEC, Saitama, Japan). The lysates were then mixed with 100 μL of chloroform and centrifuged at 12,000 × *g* for 10 minutes at 4 °C. Following centrifugation, the aqueous and organic phases were collected for total RNA and DNA isolation, respectively. Purification of RNA and DNA was performed according to the manufacturer's protocol. The concentration of RNA and DNA were assessed using a NanoDrop OneC spectrophotometer (Thermo Fisher Scientific, Waltham, MA, USA). To evaluate the structural integrity of PGN, Gram staining was conducted on LP treated with RNase, DNase, or lysozyme according to a conventional method [1], using crystal violet, fuchsin, potassium iodide, and resublimed iodine (Nacalai Tesque Inc., Kyoto, Japan).

1.3. APN activity in IEC-6 cells treated with LP-derived DNA

IEC-6 cells were seeded into 96-well culture plate at a density of 4×10^3 cells per well in test medium. After incubation for 24 h, cells were treated with DNA extracted from the untreated LP at concentrations of 342 or 684 ng/well, corresponding to the DNA yield obtained from 1×10^8 or 2×10^8 cells of LP, respectively. An equal volume of test medium was added to Control cells. After incubation for 48 h, APN activity was determined as described in Section

2.9 of the main manuscript.

2. Results

2.1. Levels of serum biochemical parameters

Levels of serum biochemical parameters are shown in Table S1. No significant differences were observed in serum levels of IgG, albumin, ketone bodies, or urea nitrogen among the aged groups. Serum NEFA concentrations were significantly lower in the EF group compared to the AC group, while no significant differences were found among the AC, LM, and LP groups.

2.2. Nucleic acids concentration and Gram staining of enzyme-treated *L. plantarum*

RNA and DNA concentrations of untreated LP, as well as LP treated with RNase, DNase, or lysozyme, are shown in Figure S1A and S1B, respectively. RNA concentration in RNase-treated LP was significantly lower than that in untreated, DNase-treated, and lysozyme-treated LP. DNA concentration in DNase-treated LP was the lowest among all treatments; however, there was no significant difference between untreated and lysozyme-treated LP. While RNase- or lysozyme-treated LP exhibited a Gram-negative staining pattern, untreated and DNase-treated LP retained a Gram-positive staining pattern (Figure S1C).

2.3. APN activity in IEC-6 cells treated with LP-derived DNA

There was no significant difference in APN activity among Control cells and cells treated with LP-derived DNA at concentrations of 342 and 682 ng (Figure S2).

Reference

[1] Murray, R., Determinative and cytological light microscopy. *Methods for general and molecular bacteriology* 1994.

Figure legends

Figure S1. (A) RNA and (B) DNA concentration of untreated, RNase-, DNase-, or lysozyme-treated *L. plantarum*. (C) Gram staining of untreated, RNase-, DNase-, or lysozyme-treated *L. plantarum*. Experiment was conducted in triplicate. Data are presented as mean ± SEM. Following analysis of variance by Bartlett's test, data were analyzed by two-tailed one-way ANOVA (equal variances) and then by post hoc multiple comparisons tests. Values without a shared letter exhibited statistically significant differences ($P < 0.05$). The scale bar represents 10 μm. Abbreviations: LP: untreated *L. plantarum*; LP (RNase): RNase-treated *L. plantarum*; LP (DNase): DNase-treated *L. plantarum*; LP (Lysozyme): lysozyme-treated *L. plantarum*.

Figure S2. APN activity of IEC-6 cells treated with DNA derived from heat-killed *Lactobacillus plantarum*. APN activity of IEC-6 cells treated with *L. plantarum* DNA at 342 or 684 ng per well. Data are presented as mean ± SEM. Following analysis of variance by Bartlett's test, data were analyzed by two-tailed one-way ANOVA (equal variances) and then by post hoc multiple comparisons tests. Abbreviations: Control: cells without treatment; LP: untreated *L. plantarum*; APN: aminopeptidase N.

Table S1 Levels of serum biochemical parameters.

	Aged mice			
	**AC**	**EF**	**LM**	**LP**
IgG (mg/mL)	0.33 ± 0.06	0.37 ± 0.11	0.47 ± 0.09	0.44 ± 0.10
Albumin (g/dL)	0.82 ± 0.05	0.82 ± 0.10	0.76 ± 0.07	0.89 ± 0.04
NEFA (mEq/L)	1.36 ± 0.10[a]	0.96 ± 0.13[b]	1.09 ± 0.09[ab]	1.21 ± 0.10[ab]
Ketone body (µmol/L)	97.23 ± 8.86	113.90 ± 51.04	118.60 ± 47.51	78.39 ± 12.36
Urea Nitrogen (mg/dL)	23.04 ± 5.28	19.55 ± 2.10	30.87 ± 6.54	24.80 ± 1.15

[1]Values are given as means ± SEMs. Values without a common letter are statistically significantly different ($P < 0.05$).

[2]One-way ANOVA or Kruskal-Wallis test followed by post-hoc test was conducted.

Abbreviations: AC: aged mice fed a Control diet; EF: aged mice fed an *Enterococcus faecalis*-supplemented diet; LM: aged mice fed a *Leuconostoc mesenteroides*-supplemented diet; LP: aged mice fed a *Lactiplantibacillus plantarum*-supplemented diet; APN: aminopeptidase N; NEFA: non-esterified fatty acid.

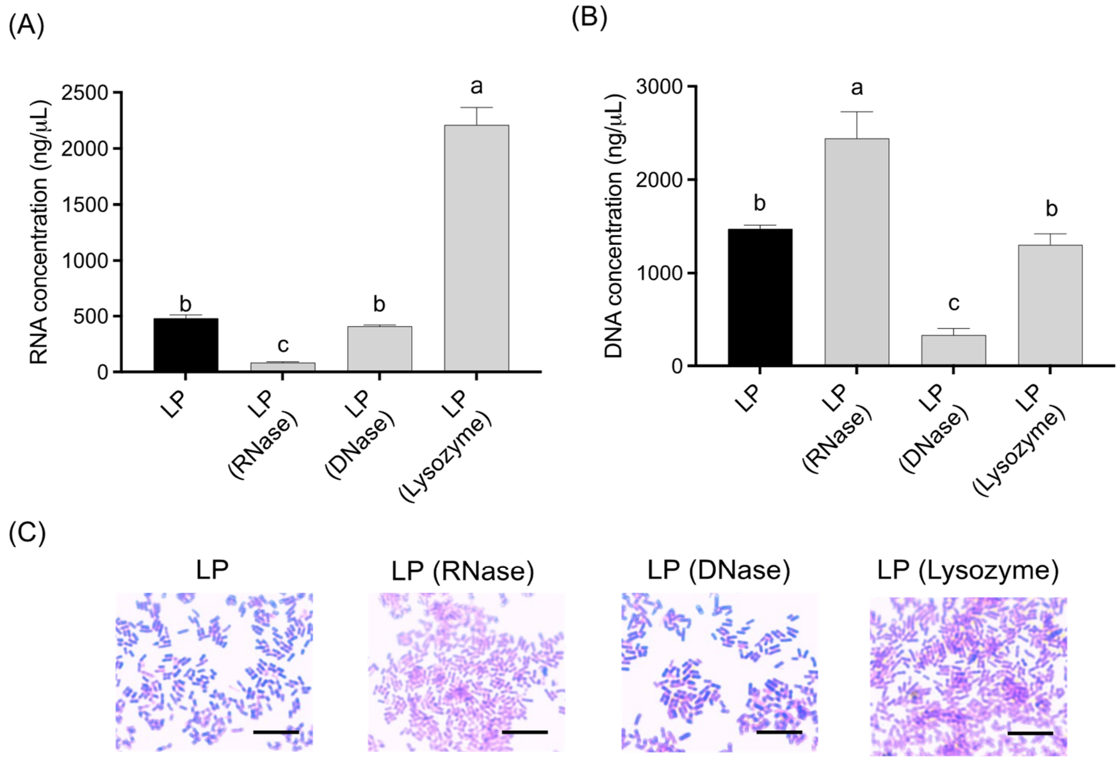
(A)
RNA concentration (ng/μL)
2500
2000
1500
1000
500
0
b
c
b
a
LP
LP (RNase)
LP (DNase)
LP (Lysozyme)
(B)
DNA concentration (ng/μL)
3000
2000
1000
0
b
a
c
b
LP
LP (RNase)
LP (DNase)
LP (Lysozyme)
(C)
LP
LP (RNase)
LP (DNase)
LP (Lysozyme)

Figure S1

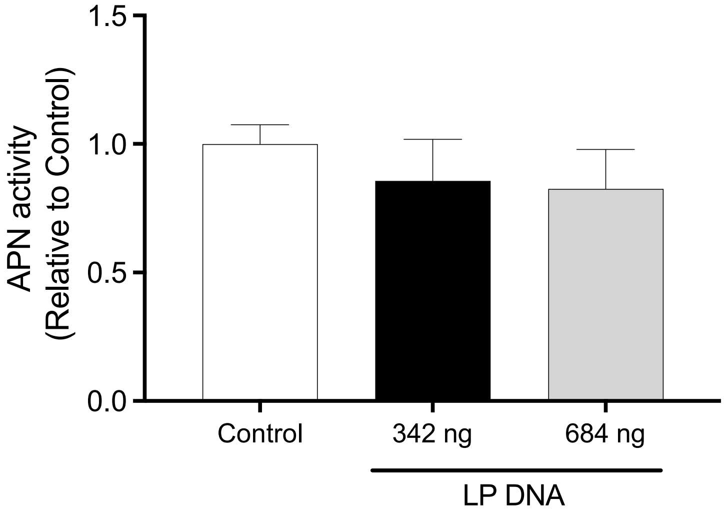
APN activity
(Relative to Control)
1.5
1.0
0.5
0.0
Control
342 ng
684 ng
LP DNA

Figure S2
